# Statin as a novel pharmacotherapy of pulmonary alveolar proteinosis

**DOI:** 10.1038/s41467-018-05491-z

**Published:** 2018-08-07

**Authors:** Cormac McCarthy, Elinor Lee, James P. Bridges, Anthony Sallese, Takuji Suzuki, Jason C. Woods, Brian J. Bartholmai, Tisha Wang, Claudia Chalk, Brenna C. Carey, Paritha Arumugam, Kenjiro Shima, Elizabeth J. Tarling, Bruce C. Trapnell

**Affiliations:** 1 Translational Pulmonary Science Center, Children’s Hospital Medical Center, Cincinnati, OH USA; 2 Division of Pulmonary Biology, Children’s Hospital Medical Center, Cincinnati, OH USA; 3 Division of Pulmonary Medicine, Children’s Hospital Medical Center, Cincinnati, OH USA; 4 Division of Pulmonary, Critical Care, and Sleep Medicine, University of Cincinnati College of Medicine, Cincinnati, OH USA; 5 Division of Pulmonary and Critical Care Medicine, University of California Los Angeles, Los Angeles, CA USA; 6 Department of Medicine, University of California Los Angeles, Los Angeles, CA USA; 7 Department of Radiology, Mayo Clinic, Rochester, MN USA; 8 Molecular Biology Institute, University of California Los Angeles, Los Angeles, CA USA

## Abstract

Pulmonary alveolar proteinosis (PAP) is a syndrome of reduced GM-CSF-dependent, macrophage-mediated surfactant clearance, dysfunctional foamy alveolar macrophages, alveolar surfactant accumulation, and hypoxemic respiratory failure for which the pathogenetic mechanism is unknown. Here, we examine the lipids accumulating in alveolar macrophages and surfactant to define the pathogenesis of PAP and evaluate a novel pharmacotherapeutic approach. In PAP patients, alveolar macrophages have a marked increase in cholesterol but only a minor increase in phospholipids, and pulmonary surfactant has an increase in the ratio of cholesterol to phospholipids. Oral statin therapy is associated with clinical, physiological, and radiological improvement in autoimmune PAP patients, and ex vivo statin treatment reduces cholesterol levels in explanted alveolar macrophages. In *Csf2rb*−/− mice, statin therapy reduces cholesterol accumulation in alveolar macrophages and ameliorates PAP, and ex vivo statin treatment increases cholesterol efflux from macrophages. These results support the feasibility of statin as a novel pathogenesis-based pharmacotherapy of PAP.

## Abstract (web summary)

Pulmonary alveolar proteinosis (PAP) is associated with defective macrophage clearance of surfactant. Here, the authors show that patients with PAP have altered cholesterol-to-phospholipid ratio in their surfactant, and that more importantly, statin therapy and reduction of cholesterol accumulation in macrophages can ameliorate PAP in both humans and mice.

## Introduction

Pulmonary surfactant is composed of 80% polar lipids, primarily phosphatidylcholine, and multiple less-abundant phospholipid species, 10% neutral lipids, primarily free cholesterol with small amounts of triglycerides and free fatty acids, and 10% surfactant proteins^1,2^ Since cholesterol content regulates the fluidity and surface tension-lowering effects of surfactant, which are critical to alveolar stability and lung function, surfactant composition is tightly regulated^3^. Surfactant homeostasis is maintained by balanced secretion by alveolar epithelial type II cells and clearance via recycling and catabolism in these cells and by catabolism in alveolar macrophages^4^. Prior studies reporting the relative fractional composition of surfactant phospholipids is normal in pulmonary alveolar proteinosis (PAP) patients^5^ and *Csf2−/−* mice^6^, led to a widely-held belief that surfactant accumulation in PAP is caused by impaired catabolism of phospholipids within alveolar macrophages^7^, however, to date, no such mechanism has been identified.

Alveolar macrophages require granulocyte/macrophage-colony-stimulating factor (GM-CSF) for maturation in the lungs and to enable surfactant clearance in vitro and in vivo^8,9^. Further, disruption of GM-CSF signaling by either GM-CSF autoantibodies as occurs in autoimmune PAP^10–12^ or by *CSF2RA* or *CSF2RB* mutations as occurs in hereditary PAP^13–15^ mediates pathogenesis in > 90% of PAP patients. PAP has no approved pharmacotherapy and is currently treated by whole lung lavage (WLL), an invasive, inefficient procedure that is repeatedly required and not widely available. Based on the observation that impaired GM-CSF-dependent cholesterol clearance within alveolar macrophages drives reduction of macrophage-mediated surfactant clearance in *Csf2rb*−/− mice^16^, a validated animal model of human PAP^17^, here, we evaluate cholesterol content in human alveolar macrophages and pulmonary surfactant from PAP patients, and test cholesterol homeostasis as a novel target for development of pharmacotherapy of PAP.

## Results

### Statin therapy and resolution of autoimmune PAP lung disease

**Fig. 1:**
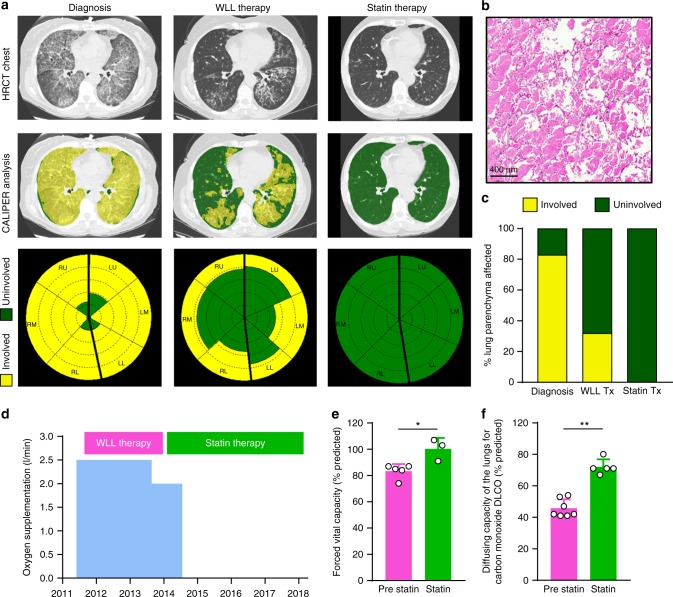
Resolution of PAP associated with oral statin therapy. **a** HRCT chest at diagnosis and after WLL therapy or statin therapy. HRCT image illustrating quantitative categorical parenchymal-pattern analysis (green-masking: uninvolved/normal lung parenchyma, yellow-masking: PAP-involved/abnormal lung comprising ground glass and reticular changes). Glyphs showing parenchymal-pattern analysis of total lung parenchyma segmented by right, left; upper, middle, and lower zones. **b** Lung histology at diagnosis. Haematoxylin and eosin. **c** Percentage of lung affected by PAP determined by parenchymal-pattern analysis. Supplemental oxygen requirement (**d**), FVC (**e**), and DLCO (**f**) before and after statin therapy. Data are mean ± SD, statistical differences determined by Student’s *t* test or Mann–Whitney test. *P* < 0.05, **P < 0.01, ***P < 0.001, ****P < 0.0001

We identified a 58-year-old woman with severe autoimmune PAP who responded poorly to WLL but improved dramatically on statin therapy. She initially presented with progressive dyspnea of insidious onset and a past medical history positive only for hypercholesterolemia. Pulmonary function testing revealed a forced vital capacity (FVC) of 74% of the predicted value and a diffusing capacity for carbon monoxide (DLCO) of 41% of predicted. A high-resolution computed tomogram (HRCT) of the chest revealed diffuse, ground glass opacification and septal thickening (Fig. 1a) and a lung biopsy identified histopathology typical of PAP (Fig. 1b). A serum GM-CSF autoantibody test^18^ was abnormal (74 mcg/ml, normal <5.0 mcg/ml) and a STAT5-phosphorylation index^13^ test indicated GM-CSF signaling was not detectable, thereby establishing a diagnosis of autoimmune PAP. Dyspnea and resting hypoxemia were treated with continuous low-flow oxygen. Multiple bilateral high-volume WLL treatments (each ~ 50 l saline/lung) were performed 1, 3, 7, 11, and 25 months after presentation with some symptomatic relief but no major effect on hypoxemia or supplemental oxygen requirement (Fig. 1d); radiographic abnormalities (Fig. 1a) and severely reduced DLCO (41–54% of predicted) persisted (Fig. 1f). GM-CSF autoantibody and STAT5-phosphorylation index tests remained abnormal at all times. Thirty-two months after presentation, statin therapy was initiated for hypercholesterolemia and lowered serum cholesterol as expected (Supplementary Table 1). Six months later, she experienced unanticipated improvement in dyspnea and elimination of her oxygen requirement (Fig. 1d), HRCT revealed reduced pulmonary ground glass opacification (Fig. 1a) and 42 months after initiating statin therapy, the abnormal PAP-related surfactant accumulation had completely resolved as determined by two different, previously validated, quantitative computed tomography (CT)-based methods (categorical parenchymal-pattern assessment (CALIPER) and densitometry^19–21^). CALIPER analysis indicated the percentage lung parenchyma affected by PAP had declined from 32% to 0% during three and half years on statin therapy (Fig. 1a, c, Supplementary Tables 2, 3). Furthermore, at this time, the FVC increased to 100% of predicted and the DLCO increased to 80% of predicted (Fig. 1e, f). Thus, initiation of statin therapy was associated with sustained clinical and radiological improvement of PAP lung disease and WLL therapy was no longer required or administered.

Subsequently, we identified a 66-year-old female with unremitting, slowly progressive autoimmune PAP (serum GM-CSF autoantibody: 109.1 mcg/ml) who had not received WLL or other therapy of PAP. Three years after diagnosis, dyspnea on exertion was persistent, the DLCO was reduced to 69% of predicted, and HRCT revealed moderate PAP (Supplementary Fig. 1a). Independently, oral statin was initiated as therapy for hypercholesterolemia; after 4 months, dyspnea had resolved and after 1 year, she remained asymptomatic, the DLCO had normalized (85% of predicted), and CALIPER analysis indicated the percentage of lung parenchyma affected by PAP had declined from 38% to 15% (Supplementary Fig. 1a, b).

### Statins reduce alveolar macrophage cholesterol in PAP

**Fig. 2:**
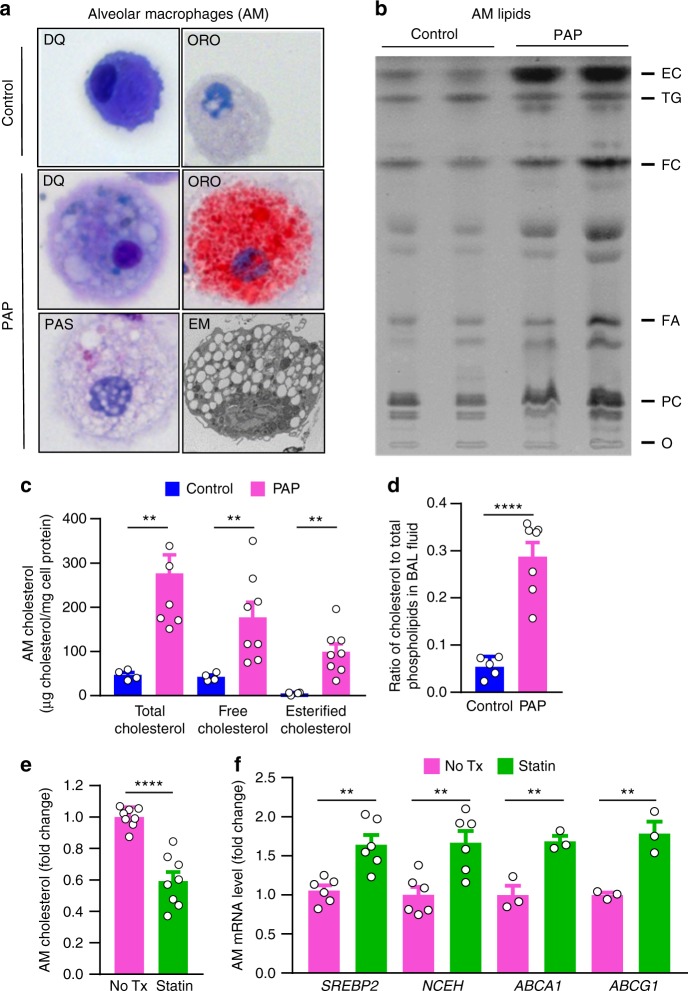
Cholesterol accumulation in human PAP alveolar macrophages and correction by statin. **a** Human alveolar macrophages (AMs) stained with Diff-Quick (DQ), Oil-red O (ORO), periodic acid–Schiff (PAS), or electron microscopy (EM). **b** Thin-layer chromatography of AM total lipids from healthy people (control) or PAP patients (PAP). **c** AM cholesterol levels in PAP or controls (*n* = 8 PAP/4 control). **d** The ratio of cholesterol to total phospholipids in pulmonary surfactant of PAP or controls (*n* = 7 PAP/5 control). **e** Total cholesterol and **f** mRNA levels for *SREBP2, NCEH, ABCA1*, *ABCG1* in PAP AMs without and after statin treatment for 24 h ex vivo (*n* = 3–6 per group). Data are mean ± SD, statistical differences determined by ANOVA with Bonferroni’s post hoc test. *P < 0.05, **P < 0.01, ***P < 0.001, ****P < 0.0001

Prompted by the clinical improvement these patients experienced on statin therapy and the recent observation that loss of GM-CSF signaling disrupts cholesterol homeostasis in *Csf2rb*−/− mice^16^, we examined the alveolar material accumulating in autoimmune PAP by studying total surfactant lipids (i.e., polar and neutral together) rather than just the polar lipid fraction as had been done previously^5^. Alveolar macrophages from autoimmune PAP patients were foamy (Fig. 2a) and contained large amounts of free and esterified cholesterol and only a small increase in phospholipids (Fig. 2b, c). Importantly, the ratio of cholesterol to phospholipids in pulmonary surfactant was markedly increased (Fig. 2d). This latter observation has physiological implications, as cholesterol regulates surfactant fluidity^22^ as well as diagnostic implications as its measurement could serve as an adjunct to the bronchoscopic evaluation of patients with ground glass opacification identified by chest CT examination.

To determine whether statin therapy may act via a direct effect on alveolar macrophages, PAP patient-derived foamy alveolar macrophages were exposed to statin therapy ex vivo for 24 hours and cholesterol content was measured. Statin treatment reduced cholesterol content by 40% compared with paired control cells (Fig. 2e). As statins inhibit 3-hydroxy-3-methylglutaryl-CoA reductase (HMGCR) thus reducing endoplasmic reticulum (ER) cholesterol levels resulting in increased expression of sterol regulatory element-binding protein-2 (*SREBP2*)^23,24^, we evaluated the effects of statin on this pathway in alveolar macrophages. Statin therapy increased expression of *SREBP2* (Fig. 2f) and its downstream target genes (Supplementary Fig. 2a) including neutral cholesterol ester hydrolase-1 (*NCEH*) (Fig. 2f), an enzyme responsible for converting esterified cholesterol to free cholesterol, which facilitates cholesterol efflux. Because ATP-binding cassette transporter family members A1 and G1 (ABCA1 and ABCG1, respectively) mediate cholesterol efflux from macrophages and their expression is abnormal in PAP^25,26^, we evaluated the effects of ex vivo statin exposure on *ABCG1/ABCA1* mRNA levels in PAP patient-derived alveolar macrophages. Statin increased mRNA transcript levels of both *ABCA1* and *ABCG1* compared to paired control cells (Fig. 2f). These results indicate statin therapy may act through a direct effect on foamy, cholesterol-laden alveolar macrophages in PAP patients by promoting cholesterol efflux.

### Statins improve PAP lung disease in *Csf2rb*−/− mice

**Fig. 3:**
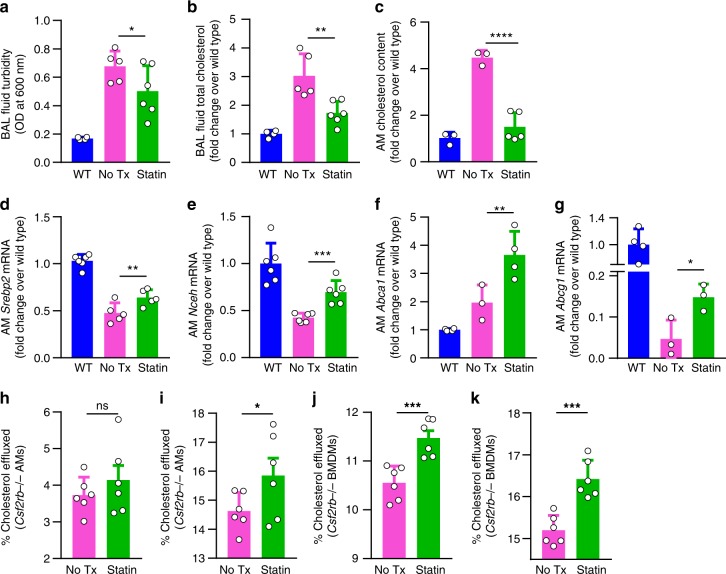
Statin therapy improves cholesterol efflux from macrophages and ameliorates PAP in *Csf2rb*−/− mice. **a**–**g** Mice received oral statin therapy for 6 weeks or untreated age-matched *Csf2rb*−/− or wild type (WT) mice. Disease severity evaluated by bronchoalveolar lavage **a** turbidity and **b** total cholesterol. **c** Alveolar macrophage cholesterol and mRNA levels for *Srebp2* **d**, *Nceh* **e***, Abca1* **f**, and *Abcg1* **g**. Cholesterol efflux capacity of *Csf2rb*−/− AMs (**h**, **i**) or BMDMs (**j**, **k**), treated with statin for 24 hours, measured by percentage of [^3^H]-cholesterol transferred to Apo-A1 (**h**, **j**) or HDL (**i**, **k**). Data are mean ± SD (3–6 mice/group), statistical differences determined by ANOVA with Bonferroni’s post hoc test. *P < 0.05, **P < 0.01, ***P < 0.001, ****P < 0.0001

Next, we asked if the clinical benefit of statin therapy could be recapitulated in vivo in a validated PAP model. To address this question, *Csf2rb−/−* mice received statin therapy by oral administration for 6 weeks. Compared with age-matched, untreated controls, statin-treated mice had reduced bronchoalveolar lavage (BAL) turbidity (Fig. 3a) (an excellent global measure of PAP sediment accumulation reflecting disease severity^17^) and reduced cholesterol levels in BAL (Fig. 3b) and alveolar macrophages (Fig. 3c) (excellent biochemical measures of PAP disease severity^17^). Similar to results for human alveolar macrophages treated with statin ex vivo, alveolar macrophages from statin-treated mice had increased mRNA transcript levels in for *Srebp2*, *Nceh*, *Abca1, and Abcg1* compared with age-matched, untreated controls (Fig. 3d–g, Supplementary Fig. 2b).

### Statins increase *Csf2rb*−/− macrophage cholesterol efflux

Because statin therapy reduced cholesterol levels in foamy alveolar macrophages from autoimmune PAP patients ex vivo and *Csf2rb*−/− mice in vivo (Figs. 2e, 3c), and cholesterol efflux is reduced in macrophages from apoE−/−GM-CSF−/− mice^27^, we measured the effects of statin on efflux of radiolabelled cholesterol from alveolar macrophages or bone marrow-derived macrophages in the presence of cholesterol acceptors—high-density lipoprotein (HDL) or apolipoprotein-A1 (Apo-A1), which preferentially accept cholesterol from macrophages via ABCG1 and ABCA1, respectively^28^. Compared with untreated paired cells, statin-treated alveolar macrophages had increased efflux in the presence of HDL but not Apo-A1 (Fig. 3h, i), whereas statin-treated bone marrow-derived macrophages (BMDMs) had increased efflux in the presence of both Apo-A1 and HDL (Fig. 3j, k). These results support the concept that statin may provide benefit as therapy of PAP by a direct effect on alveolar macrophages by increasing cholesterol clearance.

## Discussion

This study showed that cholesterol—not un-metabolized surfactant or phospholipids—was the predominant material accumulating in alveolar macrophages in PAP patients and was associated with a marked increase in the ratio of cholesterol to phospholipids in pulmonary surfactant. Statin therapy was associated with improvement in lung disease in autoimmune PAP patients, reduced cholesterol levels in alveolar macrophages from autoimmune PAP patients ex vivo, increased cholesterol efflux from *Csf2rb*−/− macrophages ex vivo, and ameliorated lung disease in *Csf2rb*−/− mice in vivo. Together, these results identify oral statin therapy as a novel pathogenesis-based pharmacotherapeutic approach for patients with autoimmune PAP.

**Fig. 4:**
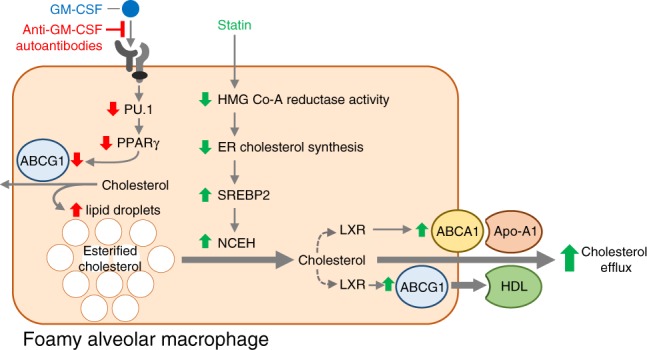
Proposed mechanisms for the pathogenesis and statin therapy of PAP. In the absence of GM-CSF signaling, surfactant-derived cholesterol accumulates progressively in lipid droplets resulting in foamy alveolar macrophages (red arrows indicate the effects of reduced GM-CSF signaling). Statin therapy results in increased cholesterol clearance from macrophages in PAP (green arrows represent the effects of statin in foamy alveolar macrophages)

The observation that abnormal accumulation of cholesterol in alveolar macrophages and pulmonary surfactant is similar in autoimmune PAP patients and in *Csf2rb*−/− mice^16^ suggests a similar mechanism may drive PAP pathogenesis. In mice, GM-CSF signaling via PU.1 is required to stimulate differentiation and functions of alveolar macrophages including surfactant clearance^8^. In humans, GM-CSF, PU.1^8^, and PPARγ^9,29^ are required for alveolar macrophage differentiation and surfactant clearance. In nonhuman primates, passive transfer of PAP patient-derived GM-CSF autoantibodies reduced expression of GM-CSF signaling axis components in alveolar macrophages in parallel with development of PAP^30^. GM-CSF is critical to cholesterol homeostasis in murine macrophages and stimulates cholesterol clearance in a constitutive, dose-dependent, and reversible fashion^16^. *Csf2rb*−/− macrophages readily clear cholesterol-deficient surfactant but clearance is reduced when cholesterol is present. Finally, *ABCA1* and *ABCG1* are vital to cholesterol homeostasis in macrophages and expression of both is abnormal in alveolar macrophages in human and murine PAP^16,25,26,31^. Further, macrophage-specific *Abcg1* gene-ablation causes PAP with accumulation of pulmonary cholesterol^32^. These results suggest a mechanism explaining the pathogenesis of autoimmune PAP (Fig. 4): reduced GM-CSF-PU.1-PPARγ axis signaling in alveolar macrophages → reduced *ABCG1/ABCA1* expression → reduced cholesterol clearance → secondarily reduced surfactant uptake and clearance (not impaired surfactant phospholipid catabolism) → surfactant accumulation in pulmonary alveoli → clinical manifestations. Several findings suggest the change in *ABCG1* expression may be more important to the pathogenesis of PAP than that of *ABCA1*, which may comprise an incompletely effective compensatory response to increased cholesterol. In *Csf2rb*−/− alveolar macrophages, *Abca1* is overexpressed while *Abcg1* is severely reduced (Fig. 3f, g) and a similar pattern seen in primary alveolar macrophages from autoimmune PAP^25^, however, both were reduced in cultured BMDMs in the absence of cholesterol exposure (albeit *Abcg1* more severely)^16^. Despite increased expression of *ABCA1* in PAP alveolar macrophages, these cells remain filled with cholesterol-rich droplets (Fig. 2a, b).

The observation that statin increased cholesterol transporter expression and efflux and reduced cholesterol levels in alveolar macrophages from PAP patients ex vivo and in *Csf2rb*−/− mice in vivo suggests a direct therapeutic effect of statins on alveolar macrophages. Indeed, statins have been reported to directly affect alveolar macrophage inflammatory cytokine release, phagocytosis, efferocytosis, and particulate clearance^33,34^. Further, statins increase cholesterol efflux from the macrophage-like cell line THP-1^35^ and activate PPARγ^36^, which is known to increase *ABCA1/ABCG1* expression and cholesterol efflux in macrohpages^25,31^. Our proposed mechanism of action of statin therapy in PAP involves inhibition of HMGCR activity leading to reduced ER cholesterol content, thus increasing *SREBP2* expression. In turn, this increases *NCEH* expression/activity^37,38^, allowing hydrolysis of cholesterol esters and liberation of cholesterol, which increases liver X receptor (LXR) activity, *ABCA1*/*G1* expression, and subsequent efflux through these transporters primarily to HDL (Fig. 4).

Our results do not exclude the possibility that effects of statin therapy on serum cholesterol and HDL may contribute to therapeutic efficacy in PAP. Indeed, autoimmune PAP patients have an increased serum LDL:HDL ratio and reduced HDL levels^39,40^ and the LDL to HDL ratio is inversely correlated with disease severity^39^. This study did not define the optimal dose of statin therapy or the time required to reach maximal treatment effect and additional studies are needed to further determine the specific mechanism of action, pharmacokinetics, and the potential role of statin therapy in autoimmune PAP. We have previously targeted cholesterol homeostasis by oral administration of PPARγ or LXR agonist therapy in *Csf2rb*−/− mice^16^. Although both statins and PPARγ agonists are available and commonly used, clinical trials will be needed to determine the relative safety and potential efficacy of these pharmacotherapies in patients with autoimmune PAP.

Finally, these results highlight the potential utility of two novel clinical outcome/diagnostic measures in the differential diagnosis of PAP. First, categorical parenchymal-pattern assessment using CALIPER software and quantitative densitometry of the lung CT scans provide continuous variable-based measures of pulmonary surfactant accumulation, which are useful in assessing PAP disease severity and may be favorable compared to semiquantitative visual assessment^41^. Second, measurement of cholesterol levels (or the cholesterol to phospholipid ratio of surfactant) in BAL could be used to evaluate patients with diffuse ground glass opacification as an adjunct to the bronchoscopic evaluation of PAP, which may reduce the need for a lung biopsy and the associated morbidity.

## Methods

### Ethical approval

The institutional review boards of the Cincinnati Children’s Hospital Medical Center and University of California Los Angeles approved this study. All human participants or their legal guardians gave written informed consent. All animal experiments were approved by the Institutional Animal Care and Use Committee (IACUC) at Cincinnati Children’s Hospital Medical Center and the Office of Animal Research Oversight (OARO) at University of California Los Angeles.

### Computed tomography densitometry analysis

A semi-automated image analysis algorithm was developed to segment the lungs and quantify the change in lung mass over serial CT images. Lung segmentation was performed using a commercially available software program (Amira, Hillsboro Oregon, USA). The whole-lungs were segmented from the body and major vasculature using an initial threshold of < 500 Hounsfield units (HU), with user oversight. The average HU values of the left and right lung were quantified along with the respective lung volumes, which were measured by summing the voxel-volumes within the lungs. Based on the average HU of each lung, mean lung tissue densities (*ρ*CT) of both left and right lungs were determined via the following equation.

$$\left( {{\mathrm{\rho CT}}} \right) = \left( {\frac{{{\mathrm{1000 - (HU)}}}}{{{\mathrm{1000}}}}} \right)$$

Studies show that lung density decreases slightly in proportion to height^42^, accordingly, a small correction was used to calculate the total lung mass (TLM) using the following formula: Predicted TLM (g)−height (cm) × 9.8759−1019.1. The data were also expressed as a percentage of the predicted TLM using the following formula: Percent error = ((TLM−Predicted TLM)/Predicted TLM) × 100). Predicted TLM was calculated from standard CT in 10 healthy control individuals (Supplementary Table 2). The patient’s predicted TLM was calculated based on the height of the patient (Supplementary Table 3).

### CALIPER parenchymal computed tomography analysis

The CALIPER (Computer-Aided Lung Informatics for Pathology Evaluation and Ratings) image analysis software was developed at Mayo Clinic (Rochester, MN, USA). CALIPER provides automated characterization and quantitative assessment of pulmonary parenchymal disease on high-resolution CT data. Comprehensive description of CALIPER methodology has been previously published^43^. In brief, data processing includes extraction of lung parenchyma from the surrounding thoracic structures (chest wall, central airways and vessels). There is additional segmentation of the lung into 12 regions (left/right with central/peripheral areas within bilateral upper/middle/lower zones) using pre-defined landmarks. Using a 15 × 15 × 15 voxel and sliding box technique, each pixel of the lung parenchyma is mapped to a parenchymal tissue type. The classification is based on the similarity of the sampled voxel histogram to the signature of regions previously determined by consensus agreement of thoracic radiologists and additional morphological characteristics. Through this process, each pixel is labeled as normal parenchyma, low attenuation areas (mild, moderate, and severe subtypes) and increased attenuation areas (ground glass opacity, reticular densities) or honeycombing. The volume of each of these characteristics and percent of total parenchyma for each feature can be quantified. For the assessment of PAP, the sum of high density features (ground glass opacity and reticular densities) was considered “Involved Lung Parenchyma” and the sum of normal and low attenuation areas was considered “Uninvolved Lung Parenchyma”. The percent involved vs. uninvolved regions was calculated based on the volume of each characteristic divided by the total segmented lung parenchymal volume.

### Human BAL and alveolar macrophages

Human BAL fluid and alveolar macrophages were obtained from BAL using flexible bronchoscopy or from discarded material of PAP patients undergoing therapeutic WLL. After the fluid was centrifuged at 283 × *g* for 10 minutes, the cellular pellet was resuspended in the culture medium. Human alveolar macrophages were isolated by adherence to tissue culture plastic. Extracellular debris was removed by gentle washing with phosphate-buffered saline (PBS). Cells were maintained in the culture medium of Dulbecco’s modified eagle’s medium (DMEM) (Life Technologies) plus 10% fetal bovine serum (FBS), 50 U/ml penicillin, and 50 µg/ml streptomycin.

### Mice used in this study

*Csf2rb* gene-deficient (*Csf2rb*^**–/–**^) mice backcrossed onto a C57BL6/J background were used for this study and the phenotype has been previously reported^44^. C57BL6/J mice (referred to as wild type or WT mice) were purchased from the Jackson Laboratory. All mice were bred and housed at the Cincinnati Children’s Research Foundation (CCRF) Vivarium or University of California Los Angeles (UCLA). Mice were maintained on a 12 hour/12 hour light/dark cycle with unlimited access to food and water. All animal experiments were approved by the IACUC at CCRF and the OARO at UCLA.

### Collection of mouse BAL fluid

Bronchoalveolar lavage (BAL) was performed in mice to acquire epithelial lining fluid and alveolar macrophages. BAL was collected from mice using five 1 ml aliquots of PBS plus 0.5 mM EDTA^17^. The 1 ml aliquots were pooled and the recovered volumes recorded. The turbidity of the fluid was measured as described below. Aliquots of the BAL were taken as pre-spun samples and total BAL lipids were extracted using chloroform and methanol. Cholesterol was measured by the Amplex red cholesterol assay, described in more detail below. Total phosphate and saturated phosphatidylcholine were measured as described below^45,46^. From the remaining BAL fluid alveolar macrophages were isolated by spinning the BAL at 280 × g for 10 minutes at 4°C to isolate a cellular pellet. The supernatant was removed and the cellular pellets were resuspended in the culture media (DMEM) for isolation of alveolar macrophages by adherence to tissue culture plastic^16^.

### Diff-Quick staining

Cells were sedimented and stained with buffered eosin and methylene blue (Diff-Quick, Fisher) and evaluated by light microscopy.

### Oil Red O staining

Cells were stained with Oil Red O staining using the Oil Red O staining kit (Poly Scientific R&D Corporation) according to the following protocol. Briefly, cells were fixed with 4% PFA and washed twice with distilled water. Cells were placed in absolute propylene glycol for 5 minutes. Propylene glycol was removed and cells were stained in a 0.5% Oil Red O solution in propylene glycol for 30 minutes. Cells were rinsed in an 85% propylene glycol solution for 5 minutes and washed twice with distilled water followed by a hematoxylin counterstain for 2 minutes. Cells were mounted with an aqueous mounting medium (glycerin jelly).

### Electron microscopy

Alveolar macrophages were collected by centrifugation (3000 rpm, 3 minutes, room temperature), incubated in modified Karnovky’s fixative (2% paraformaldehyde and 2% glutaraldehyde in 0.1 M sodium cacodylate buffer plus 0.1% calcium chloride, pH 7.3) for 2 hours, at room temperature and cell blocks were prepared and evaluated as previously described^47^.

### Lipid extraction from BAL fluid

A chloroform-methanol extraction was used to extract lipids from cells and BAL for further analysis. 1 ml of BAL was diluted in 1 ml of DPBS, subsequently 2 ml of 100% methanol, and 4 ml of 100% chloroform were added. This was mixed and then centrifuged at 4°C @1000 rpm. The lower phase containing the extracted lipids was transferred to a new glass tube for analysis.

### Cellular lipid analysis

To collect cellular lipids from primary alveolar macrophages, culture media was aspirated and then 100% isopropanol was added to the tissue culture wells. Cellular lipids were extracted for 2 hours at room temperature or overnight at 4°C. The isopropanol was then transferred into glass tubes and half the volume of new isopropanol was added back to the tissue culture plate for 30 minutes to recover any remaining sample and combined with the original volume. Following removal of isopropanol, a Pierce BCA (bicinchoninic acid assay) protein assay (Thermo Fisher Scientific) was performed on the tissue culture wells to determine the cellular protein concentration.

### Tri-one dimensional thin-layer chromatography (TOD-TLC)

Alveolar macrophages (AMs) were isolated from BALF based on adherence to tissue culture plastic as described above. Cells were repeatedly washed with PBS to remove extracellular surfactant and then 100% isopropanol was added to the tissue culture wells, 1 ml for a 12-well plate and 2 ml for a six-well plate. Cellular lipids were extracted for 2 hours at room temperature or overnight at 4°C. The isopropanol was then transferred into glass tubes and half the volume of new isopropanol was added back into the tissue culture plate for 30 minutes to recover any remaining sample and combined with the original volume. Lipid samples were then evaporated using a stream of nitrogen and a water bath set to 52°C. Cellular lipid samples were then loaded onto high performance thin-layer chromatography plates pre-coated with silica gel 60 (Fisher). Plates were prewashed with chloroform and methanol to remove any contaminants and dried overnight at 120°C. Plates were developed in a solvent system modified from White et al.^48^. In brief, plates were first developed in a Solvent mixture of chloroform, ethanol, triethylamine, and water (30:35:35:6) up to 7 cm of a 10 cm plate. Plates are removed from the chamber, dried, and placed in a second solvent of hexane and diethylether (90:10) up to 9 cm of a 10 cm plate. Plates are again removed from the chamber, dried, and then placed in the final solvent of pure hexane and run to the top of the plate. Bands are visualized by spraying with a 0.05% solution of primuline in acetone and water (80:20) and detected as ultraviolet spots at 366 nm on a Typhoon 9500 molecular imager^48^. Lipid band densities were calculated using ImageQuant Software (GE Healthcare Life Sciences) based upon standard curves.

### Cholesterol analysis

Total and free cholesterol levels were measured by fluorometric enzymatic assay as previously described^16^. Esterified cholesterol was then calculated by subtracting free cholesterol from the total value.

### BAL turbidity

The turbidity of the fluid was measured as previously described^17,49^. In brief; 250 µl of the BAL were diluted into 750 µl of PBS and the optical density measured at a wavelength of 600 nm and multiplied by the dilution factor.

### BAL phospholipid levels

Aliquots of the BAL were taken as pre-spun samples and total BAL lipids were extracted using chloroform and methanol. Total phosphate and saturated phosphatidylcholine were measured as previously reported^45,46^.

### Ex vivo human macrophage statin treatment

After purifying alveolar macrophages as described above, fresh media was added to the adherent alveolar macrophages containing human M-CSF (R&D) (25 ng/ml) only or M-CSF plus simvastatin (Calbiochem) (5 µM) and Mevalonic acid (Sigma Aldrich) (100 µM). After 24 hours of culture, cholesterol analysis or qRT-PCR was performed as described.

### RNA isolation and gene expression analysis

Total RNA was isolated using Qiazol (Life Technologies) and was converted to complementary DNA (cDNA) using the High Capacity cDNA reverse transcription kit (Applied Biosystems) according to the manufacturer’s protocol. Gene expression was determined by quantitative real-time PCR (qRT-PCR) using a Lightcycler480 Real-time qPCR machine and Lightcycler480 mastermix (Roche Diagnostics). Relative gene expression was determined using an efficiency corrected method and efficiency was determined from a 3-log serial dilutions standard curve made from cDNA pooled from all samples. Primers were designed across exon-exon boundaries (Supplementary Data 1). Results were normalized to *36b4* mRNA.

### **In vivo** statin treatment of *Csf2rb–/–* mice

For oral statin administration studies in mice, simvastatin or pravastatin was incorporated into standard rodent chow (Laboratory Rodent Diet 5001; LabDiet) at a dose expected to deliver 10 mg/kg/BW/Day (Research Diets). BAL turbidity and cholesterol levels were measured as described above. Primary alveolar macrophages were isolated and cholesterol assays and qRT-PCR were performed as described above.

### Bone marrow-derived macrophages

Bone marrow cells were obtained from 6-week old *Csf2rb–/–* mice by crushing the tibias and femurs with the culture media (DMEM (Life Technologies) plus 10% FBS, 50 U/ml penicillin, and 50 µg/ml streptomycin). Mononuclear cells were isolated by centrifugation over a Ficoll-Paque (GE Healthcare Life Sciences) gradient at room temperature for 30 minutes. The buffy coat was washed in PBS and the cellular pellet resuspended in the culture medium with M-CSF (R&D Systems) (10 ng/ml) and cells were cultured in a 10 cm dish overnight at 37°C and the next day non-adherent cells were recovered and transferred to a new dish and cultured under the same conditions for an additional 24 hrs. At this stage, non-adherent cells were discarded and adherent cells cultured for an additional 5 days to allow differentiation of BMDMs.

### Radiolabelled cholesterol efflux analysis

Alveolar macrophages or bone marrow-derived macrophages were obtained from *Csf2rb*−/− mice as described. Macrophages were plated in 24-well plates (1 × 10^5^ cells/well) in media (DMEM) containing 10% FBS and allowed to adhere for 2 h. Cells were washed and incubated for an additional 24 h in fresh media containing bovine serum albumin (BSA) (0.2% w/v), M-CSF (10 ng/ml), supplemented with an ACAT inhibitor (58-035; 2 µg/ml) and ^3^H-cholesterol (1uCi/ml). After 24 h, the cells were washed with PBS and incubated in fresh media containing 0.2% BSA and M-CSF (10 ng/ml) for a further 24 h equilibration period, with simvastatin (5 µM) and mevalonic acid (50 µM) or DMSO control. To determine cholesterol efflux, the cells were rinsed and then incubated for 4 h in media containing 0.2% BSA and M-CSF (10 ng/ml) with Apo-AI (15 µg/ml) or HDL (50 µg/ml). The media was removed, the cells washed in PBS, and the radioactive content of the media and cells determined as described^50^. Cholesterol efflux was determined by dividing the radioactive content of the media by the sum of the radioactivity in the cells and media.

### Statistical analysis

Statistical analysis was performed using GraphPad Prism 7 software. Each data point represents Mean ± SEM. For comparison of two groups, parametric (*t* test) or non-parametric (Mann–Whitney test) tests were done where appropriate. For comparison of three groups or more, statistical analysis was carried out using one-way analysis of variance, followed by Bonferroni’s post hoc test. *N* and *P* values are indicated in figure legends, and *P* values < 0.05 was considered statistically significant.

### Data availability

The authors declare that all data supporting the findings of this study are available within the paper and its supplementary information files or upon reasonable request from the authors.

## Electronic supplementary material

Supplementary Information

Description of Additional Supplementary Files

Supplementary Data 1
