# Supplementary material for: Statin as a novel pharmacotherapy of pulmonary alveolar proteinosis
Source: Nat Commun. 2018 Aug 7;9:3127. doi: 10.1038/s41467-018-05491-z (PMC6081448; doi:10.1038/s41467-018-05491-z)
Supplement: Supplementary file 1 — Supplementary Information [file 41467_2018_5491_MOESM1_ESM.pdf]

Supplementary Information

Statin as a Novel Pharmacotherapy of Pulmonary Alveolar Proteinosis.

McCarthy et al.

Supplementary Figure 1

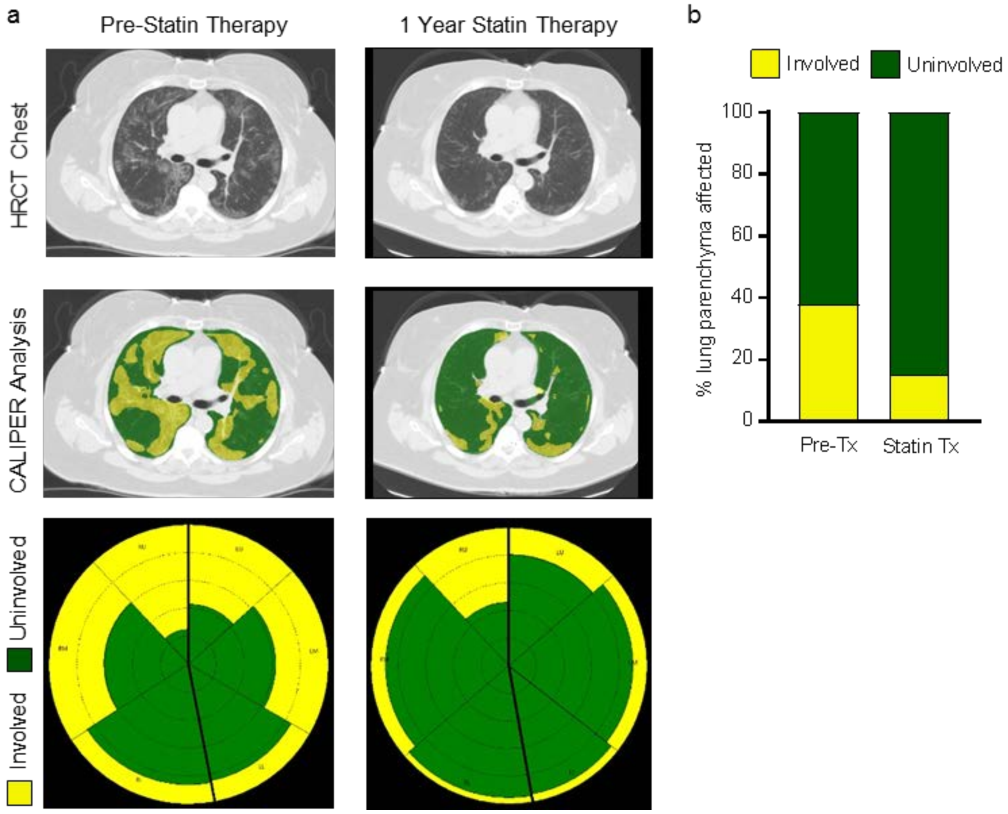

Supplementary Figure 1. a, HRCT chest images from Case 2 prior to and 1 year after initiating statin therapy. HRCT image illustrating quantitative categorical parenchymal-pattern, CALIPER analysis (green-masking: uninvolved/normal lung parenchyma, yellow-masking: PAP-involved/abnormal lung comprising groundglass and reticular changes). Glyphs showing parenchymal-pattern analysis of total lung parenchyma segmented by right, left; upper, middle, and lower zones. **b,** Percentage of lung affected by PAP determined by parenchymal-pattern analysis.

Supplementary Figure 2

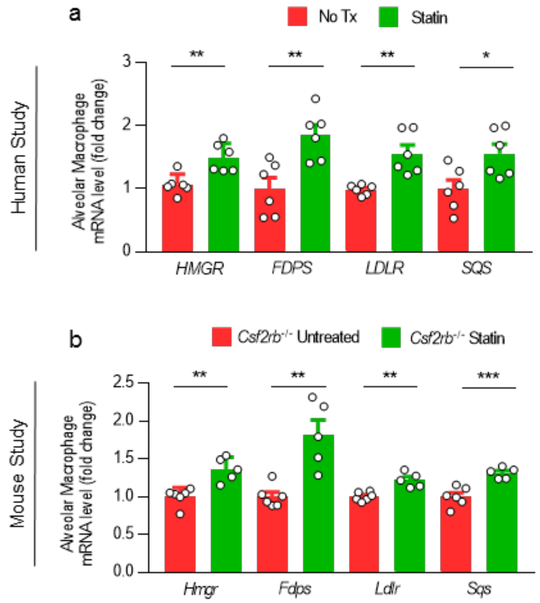

Supplementary Figure 2. Statin therapy increases expression of SREBP2 targets in alveolar macrophages (AMs). **a,** Human AM mRNA levels for *SREBP2* downstream targets: *HMGR*, *FDPS*, *LDLR* and *SQS* in PAP AMs without and after statin treatment for 24h ex vivo. **b,** Mouse AM mRNA levels for *Srebp2* downstream targets: *Hmgr*, *Fdps*, *Ldlr* and *Sqs* in AMs from *Csf2rb-/-* mice that received oral statin therapy for six weeks or untreated age-matched *Csf2rb-/-* mice. Data are Mean±SD, (n=6 per group) statistical differences determined by Student's t-test. *P<0.05,**P<0.01.***P<0.001.

Supplementary Table 1

Serum cholesterol levels of case report patient over a 10 year period (2008-2017)

Date	Total Cholesterol (mg/dL)	HDL (mg/dL)	LDL (mg/dL)	Total Cholesterol:HDL Ratio	Statin Therapy
9/13/2008	328	77	205	4.26	-
3/21/2012	214	111	87	1.93	-
2/12/2013	338	194	131	1.74	-
8/22/2013	336	45	271	7.47	-
7/20/2014	270	45	183	6.00	+
11/28/2014	265	44	192	6.02	+
2/17/2015	173	67	81	2.58	+
4/6/2015	194	68	104	2.85	+
2/23/2016	219	88	112	2.49	+
6/6/2017	175	90	72	1.94	+

Supplementary Table 2

Lung mass in healthy people determined by quantitative chest computed tomography densitometry.

Subject	Age (y)	Gender	Height (cm)	Left lung mass (g)	Right lung mass (g)	Total lung mass (g)	Predicted Total lung mass (g)*	Percent error†	Percent of predicted total lung mass‡
1	41	F	154.9	255	285	540	511	5.7	106
2	42	F	172.7	306	360	660	687	3.9	96.1
3	44	F	165.1	244	259	503	611	17.7	82.3
4	44	F	162.5	307	327	634	586	8.2	108
5	45	F	160.0	302	352	654	561	14.2	117
6	46	M	177.8	306	347	653	737	11.4	88.6
7	46	F	165.1	313	339	652	611	6.7	107
8	49	M	200.6	506	558	1064	962	10.6	111
9	54	F	172.7	313	365	678	687	1.3	98.7
10	62	M	180.3	314	349	663	762	13.0	87.0

* Predicted TLM (g) = height (cm) x 9.8759 – 1019.1

† Percent error = ((TLM – Predicted TLM) ÷ Predicted TLM) x 100

‡ Percent predicted TLM = (TLM (g) ÷ Predicted TLM) x 100

Abbreviations: TLM, total lung mass.

Supplementary Table 3

Lung mass in the patient (Case 1) determined by quantitative chest computed tomography densitometry.

Date	Left lung mass (g)	Right lung mass (g)	Total lung mass (g)	Predicted total lung mass (g)*‡	Percent of predicted total lung mass†‡
5/6/2011	727	862	1589	587	271
2/20/2012	685	685	1370	587	233
2/23/2013	497	513	1010	587	172
6/5/2017	310	351	661	587	113

* Predicted TLM (g) = height (cm) x 9.8759 – 1019.1

† Percent error -= ((TLM – Predicted TLM) ÷ Predicted TLM) x 100

‡ Median age of the control cohort (Supplementary Table 2) was younger than that of the case, however, CT measurements were normalized to body height, as described in the methods section. A marked improvement in relative lung weight was appreciable over time.
