# Supplementary material for: Statin as a novel pharmacotherapy of pulmonary alveolar proteinosis
Source: Nat Commun. 2018 Aug 7;9:3127. doi: 10.1038/s41467-018-05491-z (PMC6081448; doi:10.1038/s41467-018-05491-z)
Supplement: Supplementary file 2 — Description of Additional Supplementary Files [file 41467_2018_5491_MOESM2_ESM.pdf]

Description of Additional Supplementary Files

File Name: Supplementary Data 1

Description: Supplementary Data 1 lists the primers used for PCR in mouse and human studies in the report.
