# Supplementary material for: Statin as a novel pharmacotherapy of pulmonary alveolar proteinosis
Source: Nat Commun. 2018 Aug 7;9:3127. doi: 10.1038/s41467-018-05491-z (PMC6081448; doi:10.1038/s41467-018-05491-z)
Supplement: Supplementary file 3 — Supplementary Data 1 [file 41467_2018_5491_MOESM3_ESM.pdf]

List of Primers Used in Study

Gene Symbol	Gene Full Name	Accession No.	Forward Primer	Reverse primer
**MOUSE**				
Abca1	ATP-binding cassette, sub-family A (ABC1), member 1	NM_013454.3	atggagcagggaagaccac	gtaggccgtgccagaagtt
Abcg1	ATP-binding cassette, sub-family G, member 1	NM_009593.2	ggcctgtctgatggccgctt	agagtagctgctggcattc
Fdft1 (Sqs)	Farnesyl-diphosphate farnesyltransferase 1 (Squalene synthase)	NM_010191.3	ccaactcaatgggtctgttcct	tggcttagcaaagtcttccaact
Fdps	Farnesyl diphosphate synthase	NM_001253751.1	atggagatgggcgagttcttc	ccgacctttcccgtcaca
Hmgcr	3-Hydroxy-3-Methylglutaryl-CoA Reductase	NM_008255.2	cttgtggaatgccttgtgattg	agccgaagcagcacatgat
Ldlr	Low-density lipoprotein receptor	NM_001252659.1	aggctgtgggctccatagg	tgcggtccagggtcatct
Nceh1	Neutral cholesterol ester hydrolase 1	NM_178772.3	cagctgtgcacaacaatgg	gacctgtgggactagcttgt
Srebf2	Sterol regulatory element binding factor 2	NM_033218.1	gcgttctggagaccatgga	acaaagttgctctgaaaacaaat
**HUMAN**				
ABCA1	ATP-binding cassette, sub-family A (ABC1), member 1	NM_005502.3	tgtccagtccagtaatggttc	aagcgagatatggtccggatt
ABCG1	ATP-binding cassette, sub-family G, member 1	NM_016818.2	tggatacagggagacgggca	ctgcatgatgtagcaggaca
FDFT1 (SQS)	Farnesyl-diphosphate farnesyltransferase 1 (Squalene synthase)	NM_001287742.1	accatcatccgtgactatctg	cttcttaacatacctgctgctccaa
FDPS	Farnesyl diphosphate synthase	NM_001135821.1	tatcagaagccgggcatagg	ggtgctgtgatgaggtcgaga
HMGCR	3-Hydroxy-3-Methylglutaryl-CoA Reductase	NM_000859.2	acaataagatctgtggttgga	gctatgcatcgtgttattgtc
LDLR	Low-density lipoprotein receptor	NM_000527.4	gacgtggcgtgaacatctg	ctggcaggcaatgctttgg
NCEH1	Neutral cholesterol ester hydrolase 1	NM_020792.4	tgacagtgctggtggaaatc	tttttaggctggcatcttgagt
SREBF2	Sterol regulatory element binding factor 2	NM_004599.3	caccagctgcacatcacag	gccatgtgtacatcggaaca
